# *SDG711* Is Involved in Rice Seed Development through Regulation of Starch Metabolism Gene Expression in Coordination with Other Histone Modifications

**DOI:** 10.1186/s12284-021-00467-y

**Published:** 2021-03-05

**Authors:** Xiaoyun Liu, Junling Luo, Tiantian Li, Huilan Yang, Ping Wang, Lufang Su, Yu Zheng, Chun Bao, Chao Zhou

**Affiliations:** 1 Institute for Interdisciplinary Research, Jianghan University, Wuhan, 430056 China; 2 Key Laboratory of Three Gorges Regional Plant Genetics & Germplasm Enhancement (CTGU) /Biotechnology Research Center, China Three Gorges University, Yichang, 443002 China; 3 Key Laboratory of Biology and Genetic Improvement of Oil Crops, the Ministry of Agriculture and Rural Affairs, Oil Crops Research Institute, Chinese Academy of Agricultural Sciences, Wuhan, 430062 China; 4 Institute for Systems Biology, Jianghan University, Wuhan, 430056 Hubei China

**Keywords:** Rice, SDG711, Starch accumulation, Cooperation, Histone methylation and acetylation

## Abstract

SDG711 is a histone H3K27me2/3 transmethylase in rice, a homolog of CLF in Arabidopsis, and plays key roles in regulating flowering time and panicle development. In this work, we investigated the role of SDG711 in rice seed development. Overexpression and downregulation of *SDG711* lead to a decrease and increase in the expression level of genes related to starch accumulation, resulting in smaller seeds or even seed abortion. ChIP assay showed that SDG711-mediated H3K27me3 changed significantly in genes related to endosperm development, and SDG711 can directly bind to the gene body region of several starch synthesis genes and amylase genes. In addition, H3K4me3 and H3K9ac modifications also cooperate with H3K27me3 to regulate the development of the endosperm. Our results suggest that the crosstalk between SDG711-mediated H3K27me3 and H3K4me3, and H3K9ac are involved in starch accumulation to control normal seed development.

**Supplementary Information:**

The online version contains supplementary material available at 10.1186/s12284-021-00467-y.

## Background

Rice is one of the most important alimentary crops and provides food for more than half of the world’s population. The main body of the rice seed is the food. Starch metabolism is a key process in seed development and directly affects grain yield and quality. Starch metabolism mainly comprises starch synthesis and starch degradation.

Starch synthesis genes in the seed can be divided into four groups according to the specific tissue and developmental stage: group I genes, which are expressed during the very early periods of grain formation and are thought to be involved in the construction of fundamental cell machinery and initiation of starch granules; group II genes, which are highly expressed during endosperm development; group III genes, of which transcript levels are low at the onset but rise sharply at the start of starch synthesis in the endosperm and are presumed to play essential roles in endosperm starch synthesis; and group IV genes, which are mainly expressed at the onset of grain development with scanty expression and might be involved in synthesis of starch in the pericarp (Ohdan et al. 2005). The mechanism regulating starch biosynthesis in cereal seeds is not well understood. To date, genetic and functional genomics studies have only identified some key regulators of starch synthesis. For example, the MYC transcriptional factor OsBP-5 can interact with an ethylene-responsive element binding protein (EREBP), OsEBP-89, to enhance the transcription of *OsGBSSI* which belongs to the group III starch synthesis genes. Knockdown of OsBP-5 expression results in reduced expression of *OsGBSSI*, leading to a reduction in the amylose content of mature seeds (Zhu et al. 2003). The *FLO2* gene, encoding a nuclear-localized TPR-binding protein, has been shown to positively regulate the expression of starch synthesis-associated genes by interacting with bHLHs transcription factors (She et al. 2010). The AP2/EREBP TF gene *RSR1* negatively regulates starch synthesis genes including *OsGBSSI*, *OsSSI* and *OsSSIIIa*, In *rsr1* mutants, the expression levels of these genes are increased, while in *RSR1* overexpression, the expression of these genes is decreased (Fu and Xue 2010). It has been shown that OsbZIP58 encodes a key transcriptional regulator required for starch synthesis through directly binding to the promoters of *OsAGPL3*, *OsGBSSI*, *OsSSIIa*, *SBE1*, *OsBEIIb*, and *ISA2* to promote their expression. (Wang et al. 2013). During grain filling, starch biosynthesis genes such as *OsGBSSI*, *OsSSI*, *OsSSIIIa*, and *OsAGPL2* are up-regulated in *SERF1* knockout grains. Moreover, *SERF1* is a direct upstream regulator of *GBSSI* (Schmidt et al. 2014). In contrast to these positive and negative regulators controlling starch biosynthesis gene expression, FLO6 (a CBM domain containing protein) may act as a starch-binding protein involved in starch synthesis and compound starch grain formation through direct interaction with isoamylase1 (OsISA1) which belongs to the third group of starch synthase genes in developing rice seeds (Peng et al. 2014). In addition to genetic approaches, genome-wide DNA methylation analysis of a series of developmental stages of rice endosperm has revealed that DNA methylation is involved in the repression of genes involved in starch synthesis during seed development (Xing et al. 2015).

On the other hand, starch degradation genes mainly contain α- and β-amylases (Asatsuma et al. 2006; Zhang et al. 2016). There are few studies on the regulation mechanism of amylases. It has been shown that *SERF1* negatively regulates germination by controlling *RPBF* expression, which mediates the gibberellic acid (GA)-induced expression of *RICE AMYLASE1A* (*RAmy1A*). Loss of *SERF1* enhances RPBF expression, resulting in larger grains with increased starch content, while *SERF1* overexpression represses *RPBF*, resulting in smaller grains (Schmidt et al. 2014). OsSRT1 mediates H3K9ac by directly binding to starch metabolism genes such as *OsAmy3B*, *OsAmy3E*, *OsBmy4*, and *OsBmy9* to regulate the expression of these genes, and down-regulation of *OsSRT1* leads to abnormal seed development (Zhang et al. 2016). Therefore, the regulation of starch metabolism is crucial for determining rice yield and quality. However, the chromatin and epigenetic mechanisms that directly regulate rice starch metabolism are not well described.

Histone acetylation and methylation are important chromatin modifications that regulate gene expression during plant development (He et al. 2011). In particularly, H3 lysine 9 acetylation (H3K9ac) and H3 lysine 4 trimethylation (H3K4me3) are closely associated with genes with high expression levels and low tissue specificity, whereas histone H3 lysine 27 trimethylation (H3K27me3) is associated with genes with low expression levels and high tissue specificity. (Charron et al. 2009; Makarevitch et al. 2013; Zhang et al. 2009; Zhang et al. 2007). Polycomb group (PcG) and Trithorax-group (TrxG) proteins catalyze H3K27me3 or H3K4me3, respectively (Steffen and Ringrose 2014). Several studies have revealed that some development-related genes have bivalent modifications (H3K4me3-H3K27me3) to coordinately regulate their expression in plants (Berr et al. 2010; Sequeira-Mendes et al. 2014). Our previous study also suggested that the dynamic change in H3K27me3/H3K4me3 ratio of bivalent marking genes related to development during the SAM-to-IM transition is critical for genome-wide gene expression reprogramming in IM (Liu et al. 2015). Subsequently, readers (EBS and SHL) were identified as having the ability to recognize both H3K27me3 and H3K4me3 via their bromo-adjacent homology (BAH) and plant homeodomain (PHD) domains, which further confirmes that the genes can be coordinately regulated by different histone modifications (Qian et al. 2018; Yang et al. 2018).

Polycomb-repressive complex 2 (PRC2), a subset of the PcG proteins, has four core proteins: ENHANCER OF ZESTE [E(z)], SUPPRESSOR OF ZESTE 12 [Su(z)12], EXTRA SEXCOMBS (ESC) and P55 (Schuettengruber and Cavalli 2009). The E(z) protein catalyzes the addition of H3K27 methylation (Czermin et al. 2002). There are three E(z) homologs that catalyze the addition of H3K27me3 in Arabidopsis: CURLY LEAF (CLF), SWINGER (SWN), and MEDEA (MEA). MEA plays an important role in gametogenesis and early seed development, whereas CLF/SWN are partially redundant and function primarily in vegetative and reproductive development (Hennig and Derkacheva 2009). Homologs of E(z) (OsCLF/SDG711 and OsiEZ1/SDG718) have also been found in rice (Luo et al. 2009). They are required for H3K27me3 during rice flowering development and reproductive transition (Liu et al. 2014b; Liu et al. 2015). They may be involved in regulating seed dormancy, seedling growth, vegetative and reproductive development and seed development together with other PRC2 components in rice (Chen et al. 2017; Huang et al. 2016; Li et al. 2014; Liu et al. 2016; Nallamilli et al. 2013; Zhong et al. 2018). Previous studies have shown that during rice seed development, PRC2 complex mainly regulates the development of the embryo, endosperm, and seed coat by regulating transcription factors (Huang et al. 2016; Nallamilli et al. 2013).

In this study, we showed that both *SDG711* RNAi plants and overexpression plants produced smaller seeds. To understand the molecular mechanism, we examined the expression and histone modification of starch-related genes, and the direct association of SDG711 with those genes by ChIP assay using anti-SDG711 antibody. Our results suggest that SDG711 directly represses the expression of several starch synthesis genes and amylase genes through H3K27me3 modification, leading to impairing starch accumulation in developing seeds. In addition, H3K4me3 and H3K9ac enrichment also changed on these target genes. Our results suggest that H3K27me3 and H3K4me3 have antagonistic effects on starch synthesis genes and H3K27me3 and H3K9ac have antagonistic effects on amylase genes, respectively. The cooperation of SDG711-mediated H3K27me3 with H3K4me3 and H3K9ac is involved in starch accumulation to regulate normal seed development.

## Materials and Methods

### Plant Materials and Growing Conditions

Rice (*Oryza sativa spp japonica*) material used in this study was from the ‘DongJin’ (DJ) background, including the wild type (WT), *SDG711* (*OsCLF*) overexpression, and RNAi lines (Liu et al. 2014b). The germinated rice seedlings of all genotypes were transplanted in the field at the beginning of May and grown till the middle of August in Wuhan. To analyze developmental seeds, spikelet samples were collected daily from 1 day after pollination (DAP) to 7 DAP. The flowering spikelets were tagged using a pen mark on the lemmas. Approximately 300 developing seeds from 30 rice plants of each line were collected, and three biological repetitions were performed.

### Microscopy Analysis of Endosperm Structure

The collected spikelet samples were immediately fixed in 5% (v/v) formaldehyde, 5% (v/v) acetic acid, 45% (v/v) ethanol, and 45% (v/v) distilled, deionized water at 4 °C, followed by vacuum infiltration until the samples sank to the bottom of the container. Fixed samples were embedded using a Technovit 7100 (Heraeus Kulzer) and then cut into semi-thin sections of 1–5 μm thickness using a Leica RM2265 microtome for imaging.

### Gene Expression Analysis

Total RNA was extracted from seeds of 3DAP using TRIzol reagent (TransGen Biotech) according to the manufacturer’s protocol. Two micrograms of total RNA were reverse-transcribed to obtain cDNA by using DNase I and M-MLV Reverse Transcriptase (Invitrogen) according to the manufacturer’s instructions. Synthesized first-strand cDNA was used as a template for qRT-PCR. qRT-PCR was performed on an ABI 7900 instrument. The reactions were performed at 95 °C for 10 s, then 45 cycles of 95 °C for 5 s and 60 °C for 40 s. The disassociation curve analysis was performed as follows: 95 °C for 15 s, 60 °C for 20 s, and 95 °C for 15 s. Data were collected using the ABI 7900 sequence detection system following the manufacturer’s instructions. Relative expression levels were analyzed using the 2^-△△CT^ method (Livak and Schmittgen 2001). The rice *ACTIN1* gene was used as an internal control. The primers shown in Table S1 were designed using PRIMER EXPRESS 2.0 software (PE Applied Biosystems) to amplify 80–250 bp products.

### ChIP and Re-ChIP Assay

ChIP analysis was performed as previously described (Hu et al. 2012). Briefly, chromatin isolated from 4 g of seeds at 3 DAP was incubated with antibody-coated beads (Life technology, 10001D) overnight. After washing and elution, the products were reverse cross-linked. The products were then treated with protease K (9034, Takara), recovered, and used as a template for real-time PCR with primers listed in Table S1. Antibodies used for histone modifications were anti-H3K4me3 (ab8580, Abcam), anti-H3K27me3 (07–449, Millipore) and anti-H3K9ac (07–352, Millipore) respectively. The Antibody of SDG711 was produced by immunizing rabbits with E.coli produced full-length SDG711 protein (Liu et al. 2014b).

Tissue fixing, chromatin sonication, and IP with the first antibody in the primary ChIP procedure were performed as Described above. The first antibody was crosslinked to the beads using the fixative disuccinimidyl suberate (DSS, Pierce, 21,555). After washing with the buffers, the protein-DNA complexes were eluted from beads by ChIP Elution buffer (50mMTris-HCl pH 7.5, 10 mM EDTA, 1% SDS) for 15 min at 65 °C. The eluted chromatin was then used for the reChIP. ReChIP with the secondary antibody, elution, DNA purification were performed as described above.

### Yeast Two-Hybrid Assay

Constructs for yeast two-hybrid analysis were generated using the Matchmaker® Gold Yeast Two-Hybrid System (Clontech) vectors pGBKT7 and pGADT7, which express protein fusions to the GAL4 DNA-binding domain or transcriptional-activation domain, respectively. Full-length of cDNA inserts encoding OsCLF (SDG711) and OsiZE1 (SDG718) were introduced into pGADT7, and full-length of cDNA inserts encoding OsiZE1, OsFIE1, OsFIE2 and OsEMF2b were introduced into pGBKT7. The analysis was performed in strain AH109 carrying HIS3 and MEL1 reporters for reconstituted GAL4 activity.

### Starch Contents, Protein Content and 100-Grain Weight Determination

Fully filled grains of 30 DAP were used to measure grain quality and yield traits. The embryo and pericarp were removed from the dehulled grains, and the endosperm was ground to powder. The apparent amylose content (AAC) of the sample was measured by the iodine colorimetric method (Juliano 1971). To determine the total starch content, 50 mg of powder was washed two to three times using 80% (v/v) ethanol and then subjected to extraction using 9.2 and 4.6 M perchloric acid. The supernatant was collected and diluted to 50 mL with water. An aliquot of this solution was analyzed for starch content by the anthrone method (Turner and Turner 1960). Seed storage protein content was measured using near-infrared reflectance using an XDS Rapid Liquid Analyzer (FOSS Tecator AB, Sweden). All samples were measured using three replicates (Ge et al. 2007). The 100-grain weight was determined by counting 10 replicates of 100-grain samples independently on an electronic balance. Data are shown as the mean ± SD.

## Results

### Knockdown and Overexpression of the SDG711 Gene Leads to Abnormal Seed Development in Rice

**Fig. 1:**
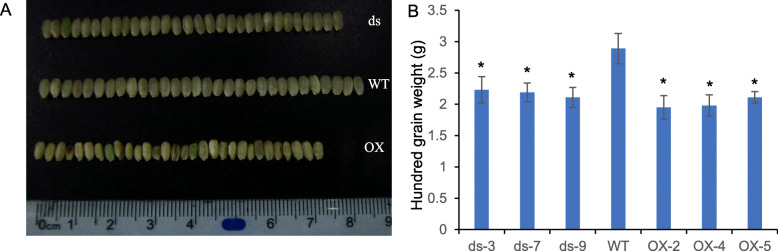
Phenotypic analysis of *SDG711* transgenic seeds. **a.** Smaller mature seeds of *SDG711* RNAi and overexpression transgenic plants. Bars = 0.5 cm. ds indicates independent lines of *SDG711* RNAi transgenic plants; OX indicates independent lines of *SDG711* overexpression transgenic plants. **b**. The 100-grain weight of *SDG711* RNAi, overexpression transgenic plants, and wild type. *significantly different (*p* < 0.01). Values are mean ± SD. ds3, ds-7 and ds-9 indicate independent lines of *SDG711* RNAi transgenic plants; OX-2, OX-4, and OX-5 indicate independent lines of *SDG711* overexpression transgenic plants

Our previous studies characterized transgenic and mutant plants for the rice E(Z) gene *SDG711* and showed that *SDG711* regulates rice flowering time and inflorescence meristem activity (Liu et al. 2014b; Liu et al. 2015). All phenotypic statistics come from three parallel lines, and all molecular biology experiments have been performed a mixture of three lines. In this study, we also observed that *SDG711* expression levels affect rice seed development. Seed setting rate was reduced by 71–89% compared to WT in both overexpression and RNAi plants (Figure S1). Through the investigation of the structure of the flowers, it was shown that the stigmas and anthers were not significantly different from WT in the external structure, whether in overexpression plants or RNAi plants. Observation of the morphology of seeds at different days after fertilization shows that seeds of overexpression plants developed significantly more slowly than WT seeds, and most of them did not develop into full seeds normally (Figure S1). Subsequently, the agronomic traits of overexpression, RNAi, and WT plants in fully mature seeds were measured. Compared with WT, the grain width and 100-grain weight of overexpression and RNAi plants were significantly decreased (Fig. 1). This indicates that SDG711 may be involved in the very complicated regulation pathways of the seed development process.

### SDG711 Affects Starch Accumulation in Endosperm Cells at the Early Stage of Seed Development

**Fig. 2:**
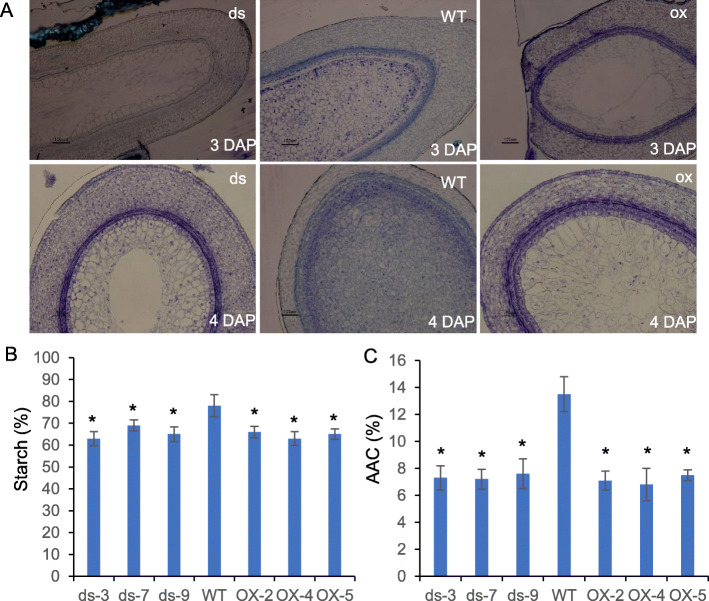
Endosperm starch granules and starch content of *SDG711* transgenic plants. **a.** Cross sections of seeds at 3 DAP and 4 DAP. ds indicates independent lines of *SDG711* RNAi transgenic plants; OX indicates independent lines of *SDG711* overexpression transgenic plants. **b.** Total starch content in endosperm of *SDG711* transgenic plants. **c.** AAC in endosperm of *SDG711* transgenic plants. *significantly different (p < 0.01). Values are mean ± SD. ds3, ds-7 and ds-9 indicate independent lines of *SDG711* RNAi transgenic plants; OX-2, OX-4 and OX-5 indicate independent lines of *SDG711* overexpression transgenic plants

Next, we examined the development of the internal structure of the seeds using semi-thin sections. The cross sections of 3 and 4 DAP seeds showed that the number of starch granules in the endosperm of overexpression plants and RNAi plants was much lower than in WT plants. In addition, the process of starch accumulation was hindered, and the starch granules are loosely distributed and cannot completely fill the cavity in the middle of the endosperm (Fig. 2a). In order to identify the mechanism related to decreased seed quality, we measured the total starch content and apparent amylose content (AAC) in mature seeds of overexpression, RNAi, and WT plants. The measurements showed that the starch content of seeds in overexpression and RNAi plants were significantly reduced by 16–19% compared to that in WT plants, while the ACC contents were reduced by 40–52% compared to that in WT plants (Fig. 2b). We also tested the content of the four main storage proteins (albumin, globulin, prolamin, and glutelin), and observed that the storage protein content in the seeds of overexpression and RNAi plants did not change significantly compared with those in WT plants (Figure S2). The above results suggest that *SDG711* may regulate seed development by playing vital roles in starch accumulation and distribution, but not in protein storage in the endosperm.

### Starch Metabolism Related Genes Were Differentially Expressed in Overexpression and RNAi Lines

**Fig. 3:**
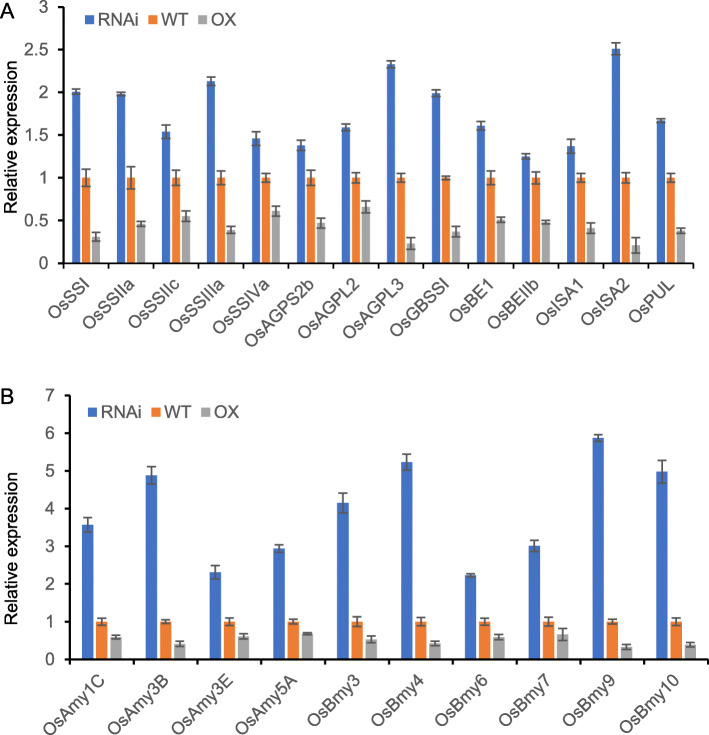
Expression level of genes associated with starch metabolism in WT and *SDG711* transgenic seeds at 3 DAP. Real-time PCR detection of transcript levels of starch synthase genes (**a**) and amylase genes (**b**) in WT and *SDG711* transgenic seeds at 3 DAP. RNAi indicates lines of *SDG711* RNAi transgenic plants and OX indicates lines of *SDG711* overexpression transgenic plants. Each qRT-PCR assay was repeated three times. Values are mean ± SD. Values are shown relative to the *ACTIN* transcript levels

The accumulation of starch in the seed endosperm is directly related to starch synthesis and starch degradation. In order to determine the causes of the low starch content in *SDG711* overexpression and RNAi lines, we analyzed the expression levels of starch metabolism-related genes in the three DAP seeds, including regulators that affect starch metabolism, starch synthesis-related genes, and starch degradation-related genes. First, we tested the typical regulator genes *RSR1*, *OsbZIP58*, *FLO2*, *FLO6*, *SERF1* and *OsBP-5* which have been found to regulate starch metabolism. However, there were no obvious differences between *SDG711* overexpression, RNAi lines, and WT plants (Figure S3). Next, we examined 24 genes of four groups of starch synthase (*OsSSI*, *OsSSIIa*, *OsSSIIb*, *OsSSIIc*, *OsSSIIIa*, *OsSSIIIb*, *OsSSIVa*, *OsSSIVb*, *OsAGPS1*, *OsAGPS2a*, *OsAGPS2b*, *OsAGPL1*, *OsAGPL2*, *OsAGPL3*, *OsAGPL4*, *OsGBSSI*, *OsGBSSII*, *OsBEI*, *OsBEIIa*, *OsBEIIb*, *OsISA1*, *OsISA2*, *OsISA3*, and *OsPUL*), 20 starch degradation genes including 10 α-amylase genes (*OsAmy1A*, *OsAmy1B*, *OsAmy1C*, *OsAmy2A*, *OsAmy3A*, *OsAmy3B*, *OsAmy3D*, *OsAmy3E*, *OsAmy4A,* and *OsAmy5A*), and 10 β-amylase genes (*OsBmy1*, *OsBmy2*, *OsBmy3*, *OsBmy4*, *OsBmy5*, *OsBmy6*, *OsBmy7*, *OsBmy8*, *OsBmy9,* and *OsBmy10*). The expression of 14 starch synthesis genes (*OsSSI*, O*sSSIIa*, *OsSSIIc*, *OsSSIIIa*, *OsSSIVa*, *OsAGPS2b*, *OsAGPL2*, *OsAGPL3*, *OsGBSSI*, *OsBEI*, *OsBEIIb*, *OsISA1*, *OsISA2*, and *OsPUL*) was higher in *SDG711* RNAi than in WT seeds, and lower in *SDG711* overexpression than in WT seeds. The degree of decrease in overexpression lines was greater than the degree of increase in RNAi lines (Fig. 3a). The expression of four α-amylase genes (*OsAmy1C*, *OsAmy3B*, *OsAmy3E*, and *OsAmy5A*) and six β-amylase genes (*OsBmy3*, *OsBmy4*, *OsBmy6*, *OsBmy7*, *OsBmy9*, and *OsBmy10*) was also higher in SDG711 RNAi than WT seeds, and lower in *SDG711* overexpression than in WT seeds. However, the degree of decrease in the overexpression lines was lower than the degree of increase in RNAi lines (Fig. 3b). Similar expression levels of other genes were detected in the overexpression, RNAi, and WT seeds (Figure S4). This result suggested that in the process of starch accumulation, the expression of starch synthesis genes was reduced by a greater extent than amylase genes, thus the starch synthesis genes may play a leading role in *SDG711* overexpressing lines, while the expression of the starch amylase genes was increased more than the starch synthesis genes, thus the starch amylase genes may play a leading role in *SDG711* RNAi lines. Therefore, we can observe that the accumulation of starch in both overexpression and knockout plants is reduced.

**Fig. 4:**
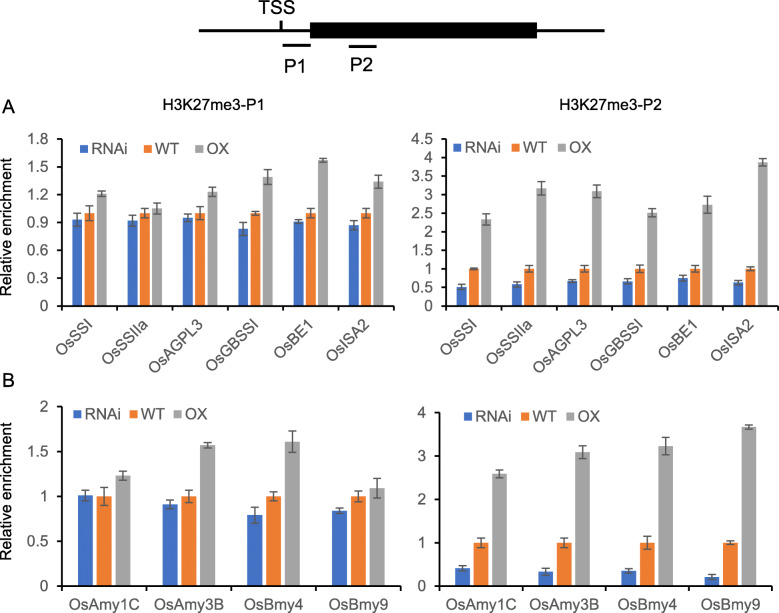
SDG711 function in H3K27me3 of starch metabolism genes. Chromatin immunoprecipitation (ChIP) analysis of H3K27me3 of starch synthase genes (**a**) and amylase genes (**b**) in WT and *SDG711* transgenic seeds at 3 DAP. H3K27me3 enrichment at the 5′ transcriptional start site (TSS) (P1) and 5′ end of the gene body (P2) was detected by quantitative PCR. Each q-PCR assay was repeated three times. Bars = mean ± SD from three technical replicates

Our previous study confirmed that SDG711 is a histone methyltransferase, that catalyzes the addition of H3K27me3 (Liu et al. 2014b). Changes in SDG711 expression can affect genome-wide H3K27me3 in rice (Liu et al. 2015). In order to study whether changes in the expression level of SDG711 would affect histone modification changes on these starch-metabolizing enzymes, ChIP assay was performed to analyze H3K27me3 on these genes in overexpression, RNAi, and WT seeds at 3 DAP. Given that H3K27me3 was enriched within gene bodies, mostly at the 5′ end of the rice genes, we analyzed ChIP by real-time PCR using two primer sets, one corresponding to the 5′ transcriptional start site (TSS), and the other to the 5′ end of the gene body (Fig. 4). Among these genes, six starch synthesis genes (*OsSSI*, *OsSSIIa*, *OsAGPL3*, *OsGBSSI*, *OsBEI*, and *OsISA2*) and four starch amylase genes (*OsAmy1C*, *OsAmy3B*, *OsBmy4*, and *OsBmy9*) displayed H3K27me3 in the gene body region, suggesting that regulation of these genes might involve PRC2 function. H3K27me3 on these genes was clearly reduced in the RNAi lines but increased in the overexpression lines (Fig. 4), which inversely correlated with their expression changes in the transgenic plants. Although the expression levels of other genes in the corresponding transgenic plants changed, the enrichment of H3K27me3 did not change significantly (Figure S5). This may support the indirect effect of SDG711 on their expression. These data suggest that SDG711-mediated H3K27me3 is involved in the regulation of starch synthesis and degradation related genes during seed development.

### SDG711 Directly Binds to Starch Synthesis Genes and Amylase Genes

**Fig. 5:**
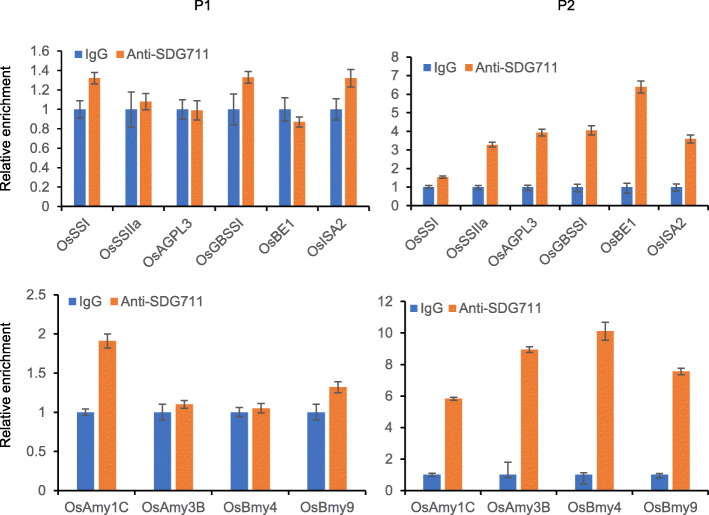
Direct association of SDG711 protein with genes of starch metabolism. SDG711 protein enrichment of the starch metabolism genes in WT seeds at 3 DAP tested by ChIP with anti-SDG711. Non-immunized serum (IgG) was used as a control. SDG711 protein enrichment at the 5′ transcriptional start site (TSS) (P1) and 5′ end of the gene body (P2) was detected by quantitative PCR (q-PCR). Each q-PCR assay was repeated three times. Values are the mean ± SD from three technical replicates

To further assess the function of SDG711 on the regulation of starch synthesis genes and amylase genes, we performed anti-SDG711 ChIP assays and analyzed them by real-time PCR using the same primer sets as for the histone methylation ChIP. Non-immunized serum was used as a control. The analysis revealed that SDG711 was obviously enhanced in the gene body of six starch synthesis genes (*OsSSI*, *OsSSIIa*, *OsAGPL3*, *OsGBSSI*, *OsBEI*, and *OsISA2*) and four starch amylase genes (*OsAmy1C*, *OsAmy3B*, *OsBmy4*, and *OsBmy9*) compared with the control (Fig. 5). These results suggest that SDG711 may directly target these genes.

### H3K27me3 Can Affect H3K4me3 and H3K9ac on some Starch Synthesis Genes and Amylase Genes

**Fig. 6:**
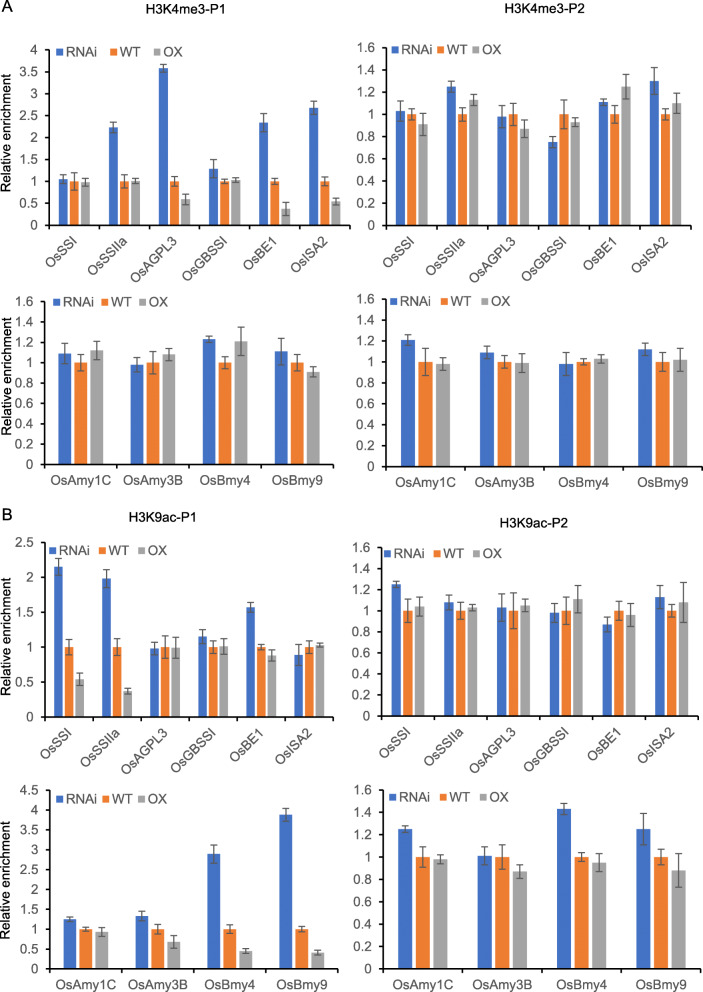
H3K4me3 and H3K9ac enrichments of starch metabolism genes. Chromatin immunoprecipitation (ChIP) analysis of H3K4me3 (**a**) and H3K9ac (**b**) of starch synthase genes and amylase genes in WT and SDG711 transgenic seeds at 3 DAP. H3K4me3 and H3K9ac enrichment at the 5′ transcriptional start site (TSS) (P1) and the 5′ end of the gene body (P2) were detected by quantitative PCR (q-PCR). Each q-PCR assay was repeated three times. Bars = mean ± SD from three technical replicates

Because H3K27me3 is antagonistic to H3K4me3 in terms of gene activity and H3K9ac has also been reported to be enriched in these genes, we analyzed whether alteration of H3K27me3 affected H3K4me3 and H3K9ac on the starch synthesis genes and amylase genes in the transgenic plants. We performed anti-H3K4me3 and H3K9ac ChIP assays and analysis by real-time PCR using the same primer sets as for H3K27me3 ChIP. Enrichment for H3K4me3 and H3K9ac occurs near the 5′ transcriptional start site (TSS), and the P1 primers were appropriate. The analysis revealed there were relatively higher levels of H3K4me3 on *OsAGPL3*, *OsBEI*, and *OsISA2b* in *SDG711* RNAi plants, and relatively lower levels in *SDG711* overexpression plants than in WT plants (Fig. 6a). This inversely correlated with the results for H3K27me3, suggesting that SDG711-mediated H3K27me3 might affect H3K4me3 at these three loci. Similarly, H3K9ac on *OsSSI*, *OsSSIIa*, *OsBmy4*, and *OsBmy9* was increased in *SDG711* RNAi plants but decreased in *SDG711* overexpression plants (Fig. 6b), which also inversely correlated with the results for H3K27me3, suggesting that SDG711-mediated H3K27me3 might affect H3K9ac at these five loci. Although the alteration of H3K27me3 is relatively obvious in other genes, the enrichment of H3K4me3 or H3K9ac on these genes did not change significantly in the transgenic plants.

**Fig. 7:**
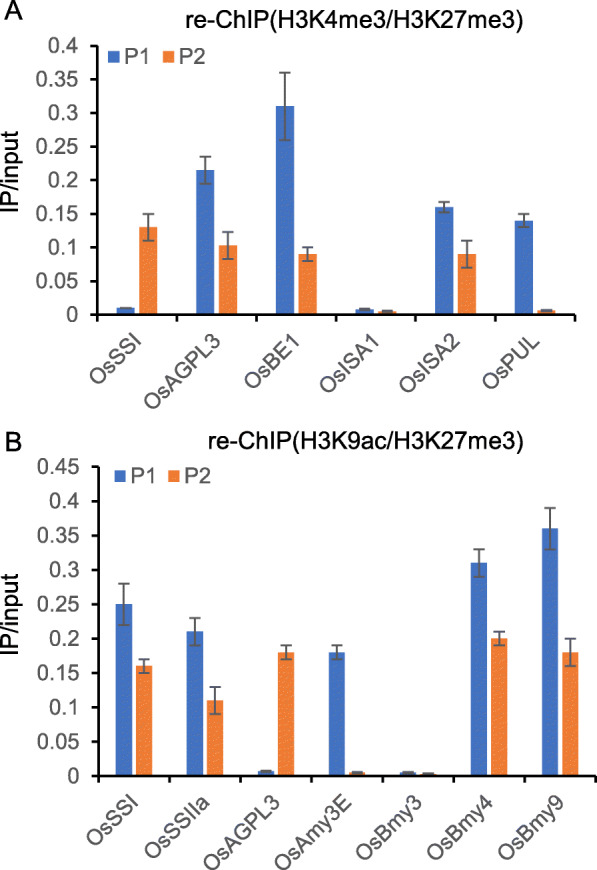
H3K4me3-H3K27me3 and H3K9ac-H3K27me3 bivalent chromatin state on some starch metabolism genes. Re-ChIP analysis of H3K4me3-H3K27me3 (**a**) and H3K9ac-H3K27me3 (**b**) of starch synthase genes and amylase genes in WT seeds at 3 DAP. H3K4me3 and H3K9ac enrichment an the 5′ transcriptional start site (TSS) (P1) and H3K27me3 enrichment at 5′ end of the gene body (P2) were detected by quantitative. Each q-PCR assay was repeated three times. Bars = mean ± SD from three technical replicates

In addition, We validated the presence of H3K4me3-H3K27me3 and H3K9ac-H3K27me3 bivalent chromatin state on some starch metabolism genes by re-ChIP (Fig. 7). The analysis revealed that there are H3K4me3-H3K27me3 bivalent chromatin state on *OsAGPL3*, *OsBEI*, and *OsISA2b* (Fig. 7a)*,* and there are H3K9ac-H3K27me3 bivalent chromatin state on *OsSSI*, *OsSSIIa*, *OsBmy4*, and *OsBmy9* (Fig. 7b)*.*

These results indicate that both single and multiple modifications can regulate gene expression, and that the multiple modifications of starch synthesis genes and amylase genes are finely balanced. Disruption of this balance causes abnormal gene expression (Figure S9).

## Discussion

In Arabidopsis, at least three PRC2-like complexes, the EMBRYONIC FLOWER (EMF), VERNALIZATION (VRN), and FERTILISATION INDEPENDENT SEED (FIS) complexes, play critical roles in different developmental stages. The EMF complex (CLF/SWN, EMF2, FIE and MSI1) mainly promotes vegetative development of the plant, and delays reproduction. The VRN complex (CLF/SWN, VRN2, FIE and MSI1) establishes epigenetic silencing of FLC after vernalization and enables flowering. The FIS complex (MEA, SWN, FIS2, FIE and MSI1) prevents seed development in the absence of fertilization and is required for normal seed development (Hennig and Derkacheva 2009). Although there have been some advances in the studies on the components of the PRC2 complex in rice (Chen et al. 2017; Folsom et al. 2014; Huang et al. 2016; Li et al. 2014; Liu et al. 2016; Liu et al. 2014b; Liu et al. 2015; Nallamilli et al. 2013; Zhong et al. 2018), the specific composition and diversity of the PRC2 complex have not been clearly described. Combining the results of previous research and our current study on the relationship between some key components (Figure S6), we propose that during the flowering stage of rice, there are two PRC2 complexes, OsCLF (SDG711)-complex and OsiZE1 (SDG718)-complex, regulating the flowering time under long daylight and short daylight, respectively. During the other development stages, the OsFIE1-complex mainly plays roles in seed development and the OsFIE2-complex plays essential roles in the regulation of rice vegetative and reproductive development (Figure S7).

MEA and FIS2 are the two core components of PRC2. It was revealed by previous studies that they are imprinted genes and only transcribed from the maternal allele in the endosperm and interact directly with each other to regulate endosperm formation by controlling the activity of a number of imprinted genes in the endosperm in Arabidopsis (Kinoshita et al. 1999; Luo et al. 2000; Spillane et al. 2000; Baroux et al. 2006). In rice, *OsFIE1*, the homolog of *FIS2* expressed only in the endosperm, is a maternally expressed imprinting gene. The other five PRC2 genes (*OsFIE2*, *OsEMF1*, *OsEMF2*, *OsCLF* and *OsiZE1*) are expressed in a wide range of tissues and are not imprinted (Luo et al. 2009). However, recent research showed that except for OsCLF (SDG711) being a non-imprinted gene, four other genes that were considered as non-imprinted genes showed the characteristics of imprinted genes at different stages of endosperm development in rice (Kuang et al. 2019). In addition, our recent research suggested that the CLF homologous gene OsCLF (SDG711) regulates endosperm development by directly binding to the gene body region of several starch synthesis genes and amylase genes to mediate H3K27me3 enrichment in rice. Meanwhile, we tested the expression of some imprinted genes surveyed previously (Luo et al. 2011; Rodrigues et al. 2013; Yuan et al. 2017) in *SDG711* overexpression plants. With the overexpression of SDG711, the expression of these genes increased or decreased, which indicates that SDG711 may also be able to regulate the development of endosperm by affecting the expression of these imprinted genes (Figure S8). Overall, although the PRC2 genes are highly conserved and play an important role in the entire growth and development stage of plants, the components, and functions of PRC2 are very different between Arabidopsis and rice such as the imprinting effect and their roles in regulating endosperm development.

The N-terminal tails of the core histones undergo multiple covalent modifications. The histone code hypothesis suggests that multiple histone modifications act in a combinatorial manner to affect gene transcription (Strahl and Allis 2000; Schreiber and Bernstein 2002). As a combination of dense marks in short clusters, situated at strategic locations in the histones, the “modification cassette” was proposed to clarify the mechanism that may control the biological reading of different modification patterns (Fischle et al. 2003). The modification cassette also indicated that there are multiple modifications on the same nucleosome to coordinately regulate the expression of the same gene. For example, the crosstalk between serine phosphorylation and lysine methylation of the mitochondrial protein DAM1 and the transcription factor p53 has been described in human cancer cells (Fischle et al. 2003; Zhang and Dent 2005). In Arabidopsis, the regulation of the FLC locus provides a plant model of how multiple chromatin-modifying systems have emerged as important components in the control of a major developmental switch, the transition to flowering. H3K4me3 and histone acetylation are associated with active FLC expression, whereas histone deacetylation and H3K9me2 and H3K27me3 are involved in FLC repression (He and Amasino 2005).

Subsequently, the regions harboring both repressive and active chromatin modifications were defined as bivalent domains (Bernstein et al. 2006). The bivalent domain (BD) marked by H3K27me3 and H3K4me3 which are catalyzed by specific PcG and TrxG complexes, respectively, was first discovered and characterized in mouse embryonic stem cells (ESCs) (Bernstein et al. 2006). The co-occurrence of H3K4me3 and H3K27me3 is often found in promoter regions of developmentally expressed TFs and developmental genes to be rapidly switched on during differentiation in specific cell types in mouse ESCs and human ESCs (Zhao et al. 2007; Bernstein et al. 2006; Xiang et al. 2020). In addition to the bivalent promoter described above, there is another class of bivalent region called a bivalent enhancer (Blanco et al. 2020). It has been shown that the co-occurrence of H3K4me1 and H3K27me3 marks the presence of bivalent enhancers in hESCs and mESCs. They likely play a key role during differentiation, similar to bivalent promoters (Rada-Iglesias et al. 2011; Zentner et al. 2011). Recent studies have shown that DNA methylation can affect the H327me3: H3K4me3 ratio of the bivalent promoter in different cell types, and DNA hypermethylation of the bivalent promoter in cancer is related to the H3K27me3: H3K4me3 ratio in embryonic stem cells (Dunican et al. 2020). In plants, there are also a few studies describing the existence of divalent modifications (H3K4me3/ H3K27me3) during the development process and stress treatment (Berr et al. 2010; Liu et al. 2014a; Sequeira-Mendes et al. 2014; Zeng et al. 2019).

Modification cassettes and bivalent modifications indicate that histone modifications play a very important role in the development of organisms, and these modifications have balanced relationships. When the balance of these modifications is disrupted, the growth and development of individual plants may be seriously affected. This study also pointed out that during the development of rice endosperm starch, some key genes might be coordinately regulated by multiple histone modifications. For example, some starch metabolism-related genes are regulated by H3K27me3 and H3K4me3, and some are regulated by H3K27me3 and H3K9ac. Under normal circumstances, these modifications will be in a balanced state. Once a certain modification changes, the balance will be broken and the gene expression will be abnormal (Figure S9). This indicates that gene expression is regulated by multiple modifications in many cases, and this regulation might be very precise.

## Conclusions

Collectively, our results show that overexpression and downregulation of SDG711 leads to a decrease and increase in the expression level of genes related to starch accumulation, resulting in smaller seeds or even seed abortion. The ChIP assay showed that SDG711-mediated H3K27me3 changed significantly in genes related to endosperm development, and SDG711 can directly bind to the gene body region of several starch synthesis genes and amylase genes. In addition, H3K4me3 and H3K9ac modifications also cooperate with H3K27me3 to regulate the development of the endosperm. Our results suggest that the crosstalk between SDG711-mediated H3K27me3 and H3K4me3, and H3K9ac are involved in starch accumulation to control normal seed development. Our work provides new insights into the regulation of endosperm development.

## Supplementary Information

**Additional file 1: Figure S1.** Seed setting rate of SDG711 transgenic plants and morphology of floral organ morphology. **Figure S2.** Storage protein content of SDG711 transgenic and WT seeds. **Figure S3.** The expression level of regulator genes of starch metabolism in WT and SDG711 transgenic seeds at 3 DAP. **Figure S4.** The expression level of some starch synthase genes and amylase genes in WT and *SDG711* transgenic seeds at 3 DAP. **Figure S5.** chromatin immunoprecipitation (ChIP) analysis of H3K27me3 of some starch synthase genes and amylase genes in WT and *SDG711* transgenic seeds at 3 DAP. **Figure S6.** Protein interactions among PRC2 members in rice. **Figure S7.** PRC2-like complexes act at different stages of the rice life cycle. **Figure S8.** The expression level of imprinted genes in WT and SDG711 transgenic seeds at 3 DAP. **Figure S9.** Balance of multiple modifications on starch metabolism genes. (PPTX 37828 kb)

**Additional file 2: Table S1.** Oligonucleotides primers used in the study.

## Data Availability

The data sets supporting the conclusions of this article are included within the article and its additional files.

## Abbreviations

H3K27me3: H3 lysine 27 trimethylation
H3K4me3: H3 lysine 27 trimethylation
H3K9ac: H3 lysine 9 acetylation
PcG: Polycomb group
OsAmy: α-amylase
OsBmy: β-amylase
ds: RNAi plants of SDG711
OX: Overexpression plants of SDG711
WT: Wildtype
DAP: Day After Pollination
ACC: Apparent Amylase Content
ChIP: Chromatin immunoprecipitation assay
qRT-PCR: Quantitative Reverse Transcription Polymerase Chain Reaction
