# Supplementary material for: SDG711 Is Involved in Rice Seed Development through Regulation of Starch Metabolism Gene Expression in Coordination with Other Histone Modifications
Source: Rice (N Y). 2021 Mar 5;14:25. doi: 10.1186/s12284-021-00467-y (PMC7936014; doi:10.1186/s12284-021-00467-y)
Supplement: Supplementary file 1 — Additional file 1: Figure S1. Seed setting rate of SDG711 transgenic plants and morphology of floral organ morphology. Figure S2. Storage protein content of SDG711 transgenic and WT seeds. Figure S3. The expression level of regulator genes of starch metabolism in WT and SDG711 transgenic seeds at 3 DAP. Figure S4. The expression level of some starch synthase genes and amylase genes in WT and SDG711 transgenic seeds at 3 DAP. Figure S5. chromatin immunoprecipitation (ChIP) analysis of H3K27me3 of some starch synthase genes and amylase genes in WT and SDG711 transgenic seeds at 3 DAP. Figure S6. Protein interactions among PRC2 members in rice. Figure S7. PRC2-like complexes act at different stages of the rice life cycle. Figure S8. The expression level of imprinted genes in WT and SDG711 transgenic seeds at 3 DAP. Figure S9. Balance of multiple modifications on starch metabolism genes. (PPTX 37828 kb) [file 12284_2021_467_MOESM1_ESM.pptx]

## Slide 1
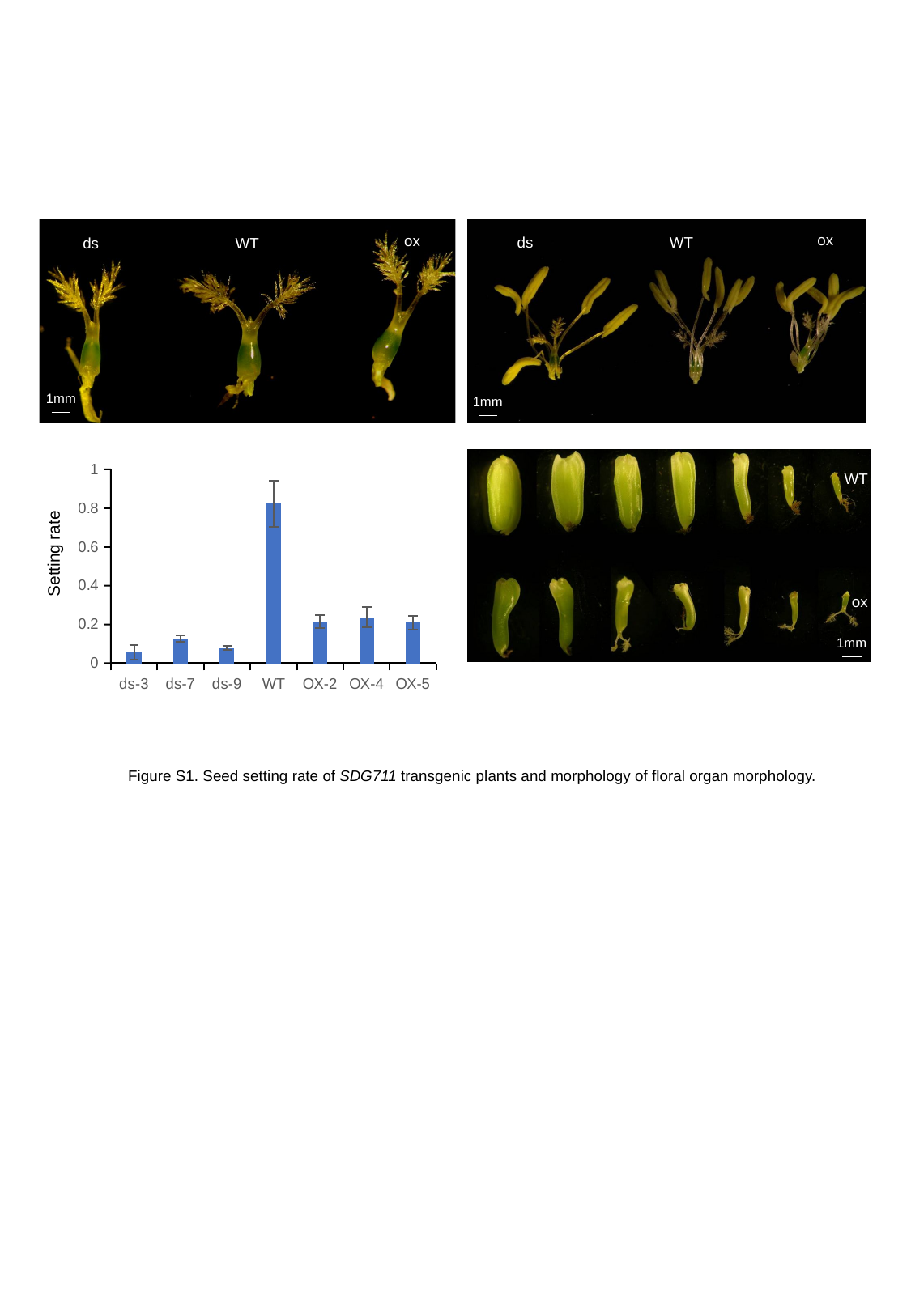

ox
ox
ds
WT
ds
WT
1mm
1mm
### Chart
| Category | |
|---|---|
| ds-3 | 0.057 |
| ds-7 | 0.127 |
| ds-9 | 0.079 |
| WT | 0.823 |
| OX-2 | 0.215 |
| OX-4 | 0.238 |
| OX-5 | 0.209 |Setting rate
WT
ox
1mm
Figure S1. Seed setting rate of SDG711 transgenic plants and morphology of floral organ morphology.

## Slide 2
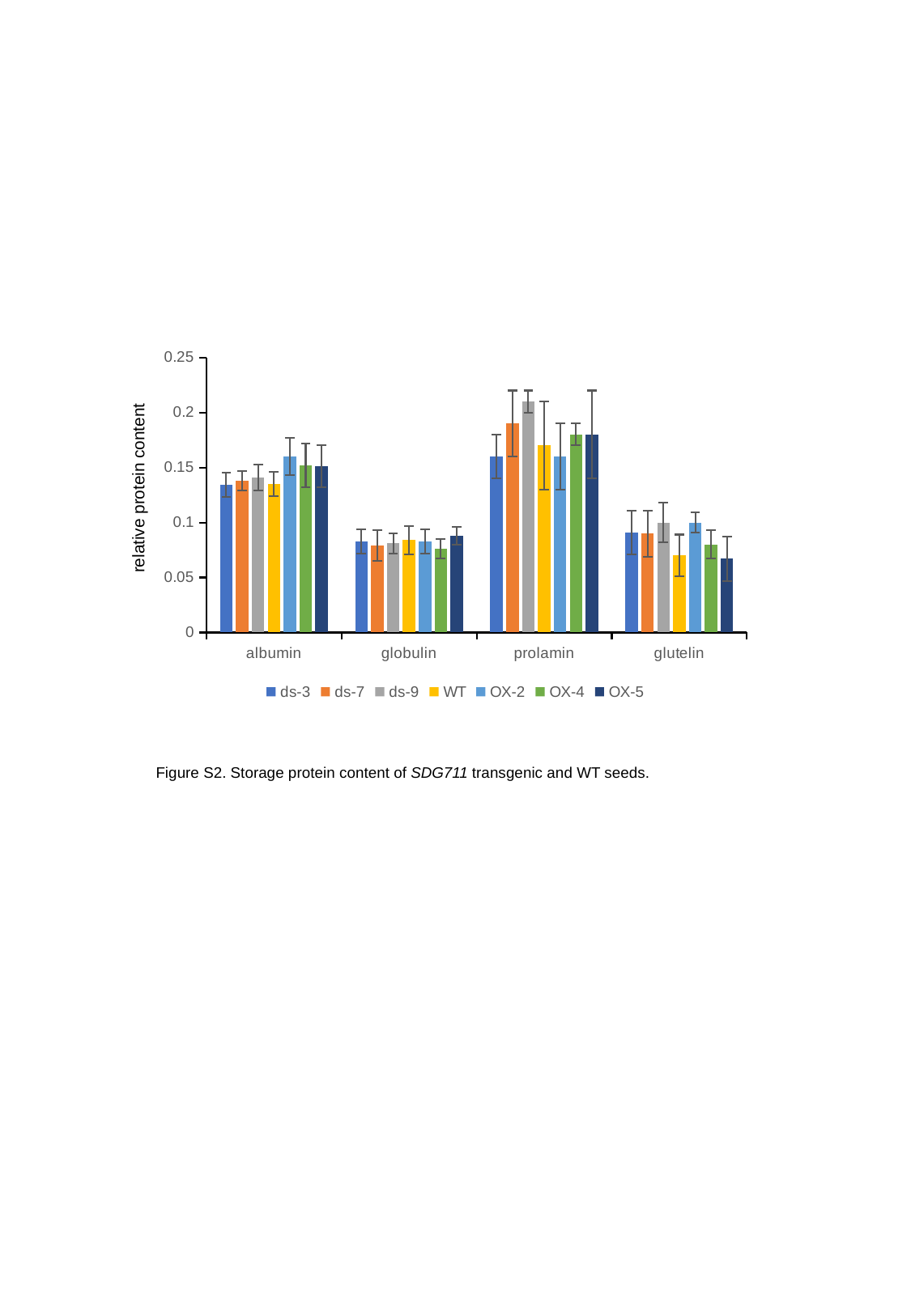

### Chart
| Category | ds-3 | ds-7 | ds-9 | WT | OX-2 | OX-4 | OX-5 |
|---|---|---|---|---|---|---|---|
| albumin | 0.134 | 0.138 | 0.141 | 0.135 | 0.16 | 0.152 | 0.151 |
| globulin | 0.083 | 0.079 | 0.081 | 0.084 | 0.083 | 0.076 | 0.088 |
| prolamin | 0.16 | 0.19 | 0.21 | 0.17 | 0.16 | 0.18 | 0.18 |
| glutelin | 0.091 | 0.09 | 0.1 | 0.07 | 0.1 | 0.08 | 0.067 |relative protein content
Figure S2. Storage protein content of SDG711 transgenic and WT seeds.

## Slide 3
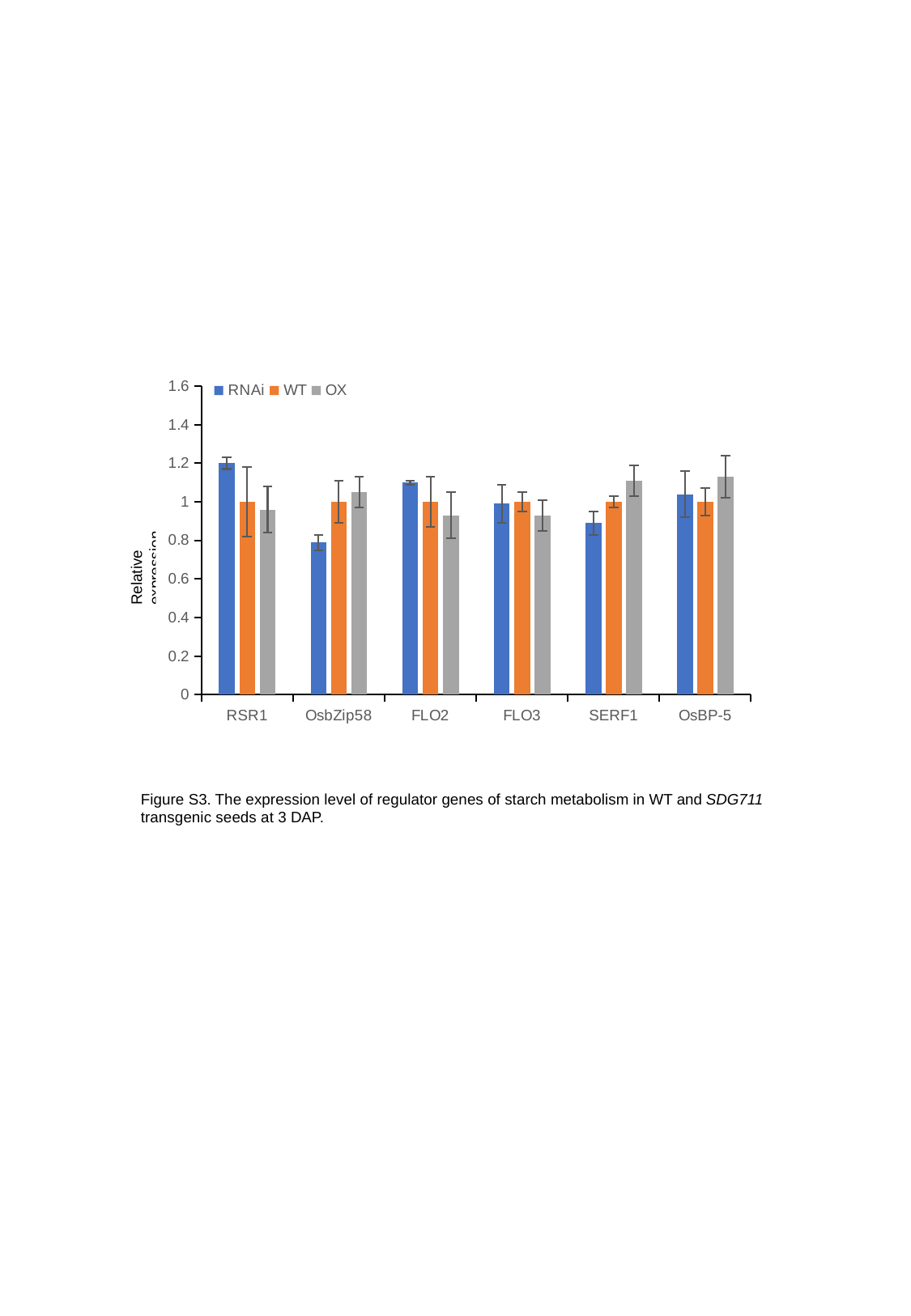

### Chart
| Category | RNAi | WT | OX |
|---|---|---|---|
| RSR1 | 1.2 | 1.0 | 0.96 |
| OsbZip58 | 0.79 | 1.0 | 1.05 |
| FLO2 | 1.1 | 1.0 | 0.93 |
| FLO3 | 0.99 | 1.0 | 0.93 |
| SERF1 | 0.89 | 1.0 | 1.11 |
| OsBP-5 | 1.04 | 1.0 | 1.13 |Relative expression
Figure S3. The expression level of regulator genes of starch metabolism in WT and SDG711 transgenic seeds at 3 DAP.

## Slide 4
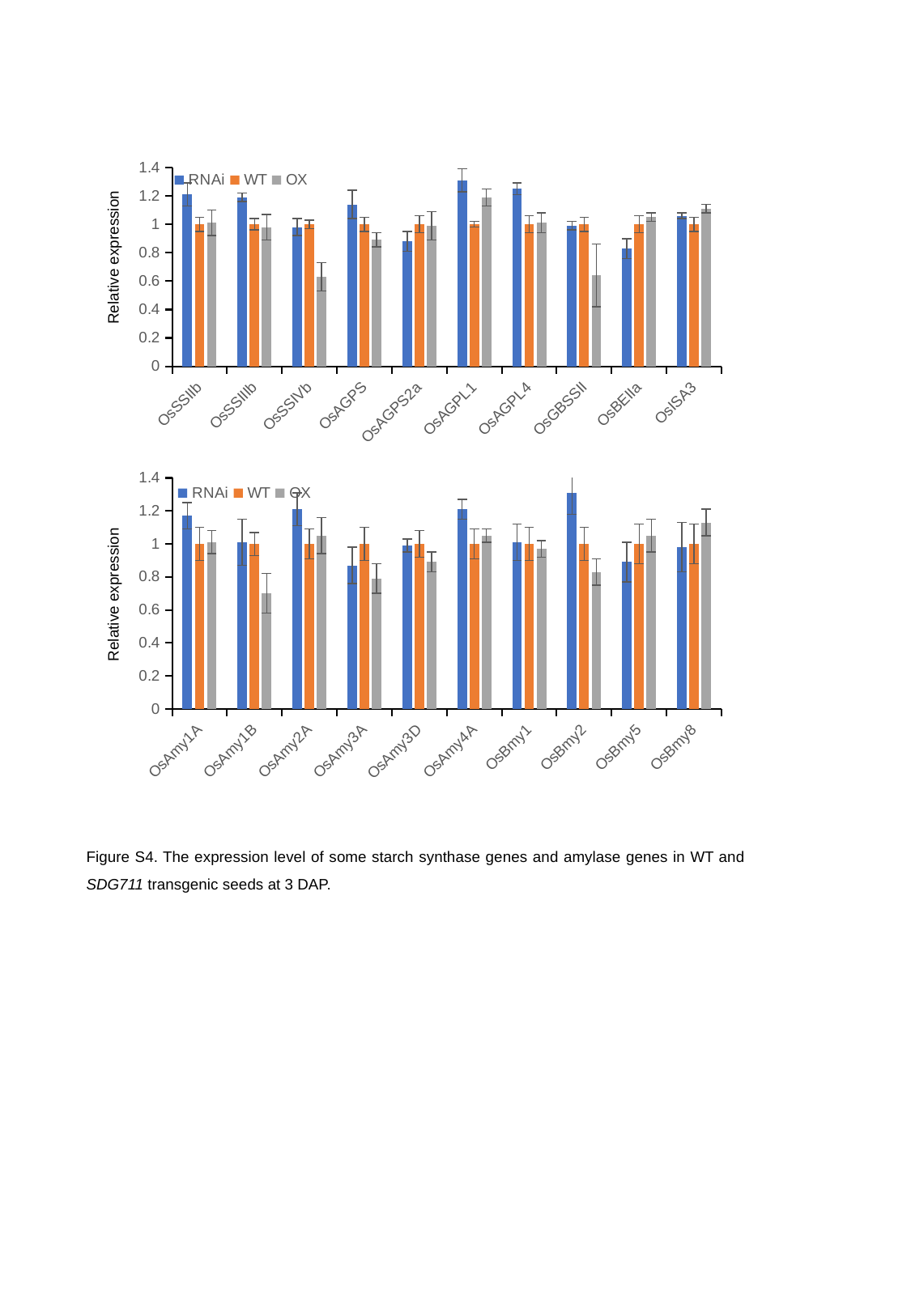

### Chart
| Category | RNAi | WT | OX |
|---|---|---|---|
| OsSSIIb | 1.21 | 1.0 | 1.01 |
| OsSSIIIb | 1.19 | 1.0 | 0.98 |
| OsSSIVb | 0.98 | 1.0 | 0.63 |
| OsAGPS | 1.14 | 1.0 | 0.89 |
| OsAGPS2a | 0.88 | 1.0 | 0.99 |
| OsAGPL1 | 1.31 | 1.0 | 1.19 |
| OsAGPL4 | 1.25 | 1.0 | 1.01 |
| OsGBSSII | 0.99 | 1.0 | 0.64 |
| OsBEIIa | 0.83 | 1.0 | 1.05 |
| OsISA3 | 1.06 | 1.0 | 1.11 |Relative expression
### Chart
| Category | RNAi | WT | OX |
|---|---|---|---|
| OsAmy1A | 1.17 | 1.0 | 1.01 |
| OsAmy1B | 1.01 | 1.0 | 0.7 |
| OsAmy2A | 1.21 | 1.0 | 1.05 |
| OsAmy3A | 0.87 | 1.0 | 0.79 |
| OsAmy3D | 0.99 | 1.0 | 0.89 |
| OsAmy4A | 1.21 | 1.0 | 1.05 |
| OsBmy1 | 1.01 | 1.0 | 0.97 |
| OsBmy2 | 1.31 | 1.0 | 0.83 |
| OsBmy5 | 0.89 | 1.0 | 1.05 |
| OsBmy8 | 0.98 | 1.0 | 1.13 |Relative expression
Figure S4. The expression level of some starch synthase genes and amylase genes in WT and SDG711 transgenic seeds at 3 DAP.

## Slide 5
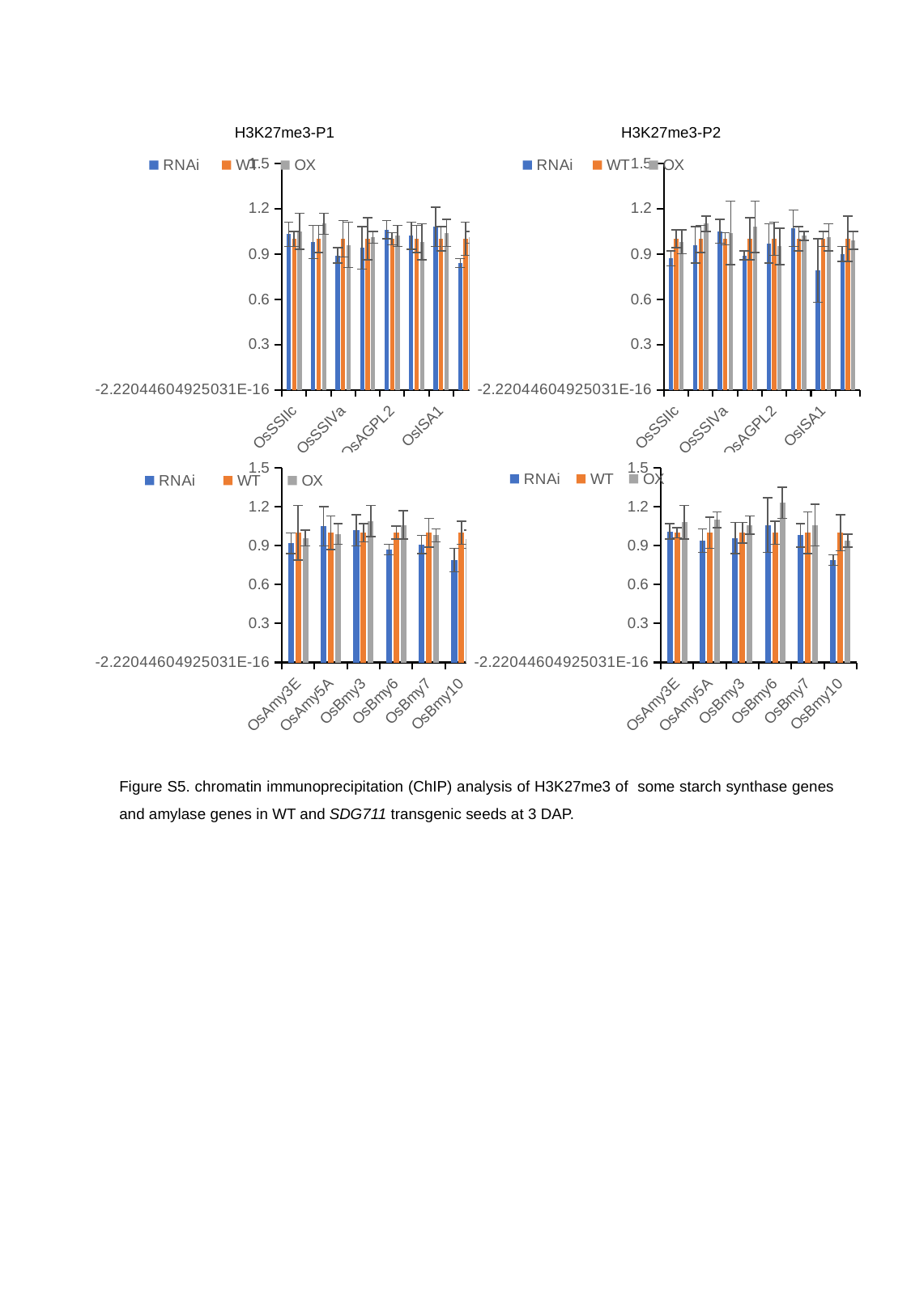

H3K27me3-P1
H3K27me3-P2
### Chart
| Category | RNAi | WT | OX |
|---|---|---|---|
| OsSSIIc | 0.87 | 1.0 | 0.98 |
| OsSSIIIa | 0.96 | 1.0 | 1.1 |
| OsSSIVa | 1.05 | 1.0 | 1.04 |
| OsAGPS2b | 0.89 | 1.0 | 1.08 |
| OsAGPL2 | 0.97 | 1.0 | 0.95 |
| OsBEIIb | 1.07 | 1.0 | 1.02 |
| OsISA1 | 0.79 | 1.0 | 1.01 |
| OsPUL | 0.9 | 1.0 | 0.99 |
### Chart
| Category | RNAi | WT | OX |
|---|---|---|---|
| OsSSIIc | 1.03 | 1.0 | 1.05 |
| OsSSIIIa | 0.98 | 1.0 | 1.1 |
| OsSSIVa | 0.89 | 1.0 | 0.96 |
| OsAGPS2b | 0.94 | 1.0 | 1.01 |
| OsAGPL2 | 1.06 | 1.0 | 1.02 |
| OsBEIIb | 1.02 | 1.0 | 0.98 |
| OsISA1 | 1.08 | 1.0 | 1.04 |
| OsPUL | 0.84 | 1.0 | 1.01 |
### Chart
| Category | RNAi | WT | OX |
|---|---|---|---|
| OsAmy3E | 1.01 | 1.0 | 1.08 |
| OsAmy5A | 0.94 | 1.0 | 1.1 |
| OsBmy3 | 0.96 | 1.0 | 1.06 |
| OsBmy6 | 1.06 | 1.0 | 1.23 |
| OsBmy7 | 0.98 | 1.0 | 1.06 |
| OsBmy10 | 0.79 | 1.0 | 0.94 |
### Chart
| Category | RNAi | WT | OX |
|---|---|---|---|
| OsAmy3E | 0.92 | 1.0 | 0.96 |
| OsAmy5A | 1.05 | 1.0 | 0.99 |
| OsBmy3 | 1.02 | 1.0 | 1.09 |
| OsBmy6 | 0.87 | 1.0 | 1.06 |
| OsBmy7 | 0.91 | 1.0 | 0.98 |
| OsBmy10 | 0.79 | 1.0 | 0.95 |Figure S5. chromatin immunoprecipitation (ChIP) analysis of H3K27me3 of some starch synthase genes and amylase genes in WT and SDG711 transgenic seeds at 3 DAP.

## Slide 6
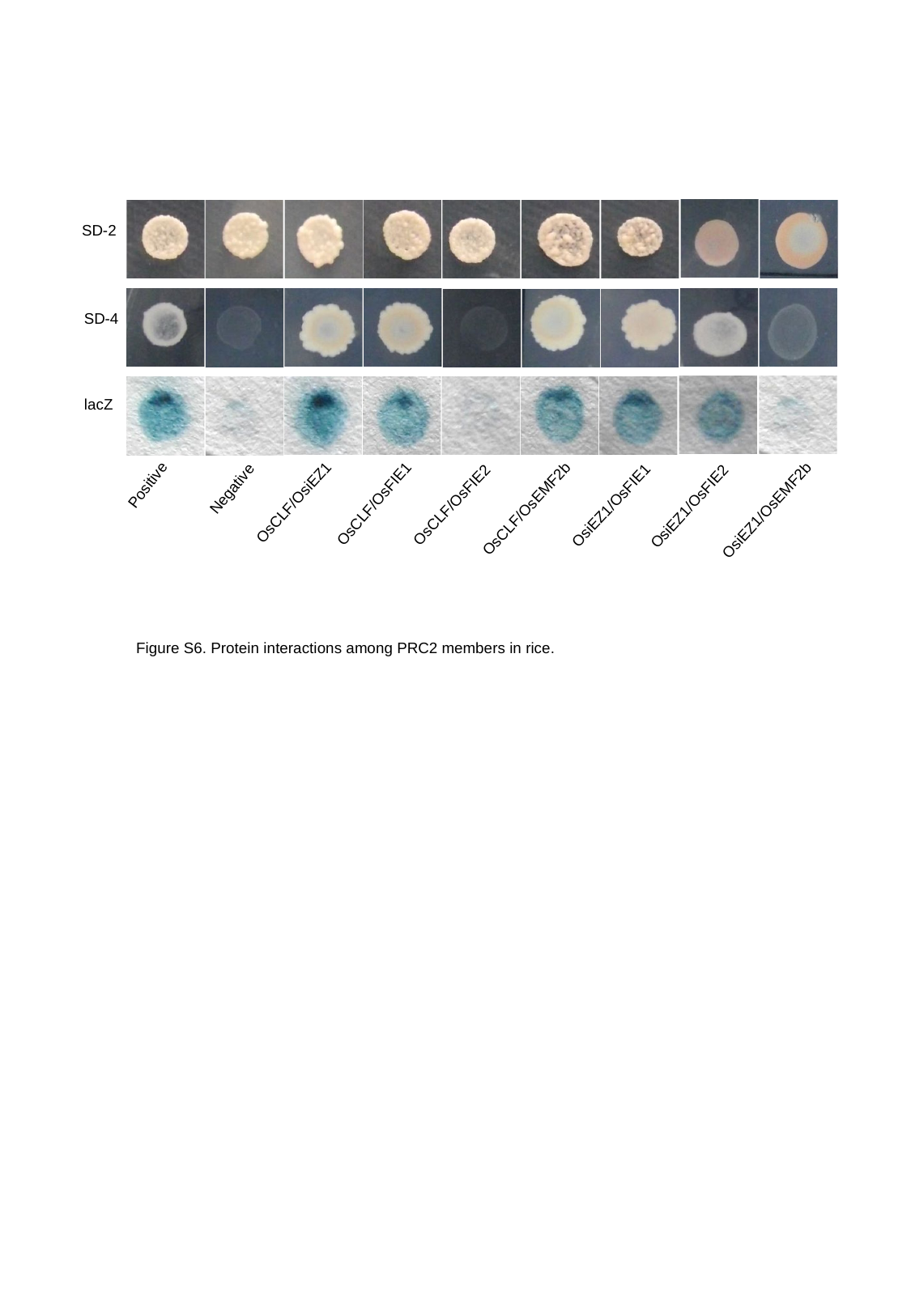

SD-2
SD-4
lacZ
Positive
Negative
OsCLF/OsiEZ1
OsCLF/OsFIE1
OsCLF/OsFIE2
OsiEZ1/OsFIE1
OsiEZ1/OsFIE2
OsCLF/OsEMF2b
OsiEZ1/OsEMF2b
Figure S6. Protein interactions among PRC2 members in rice.

## Slide 7
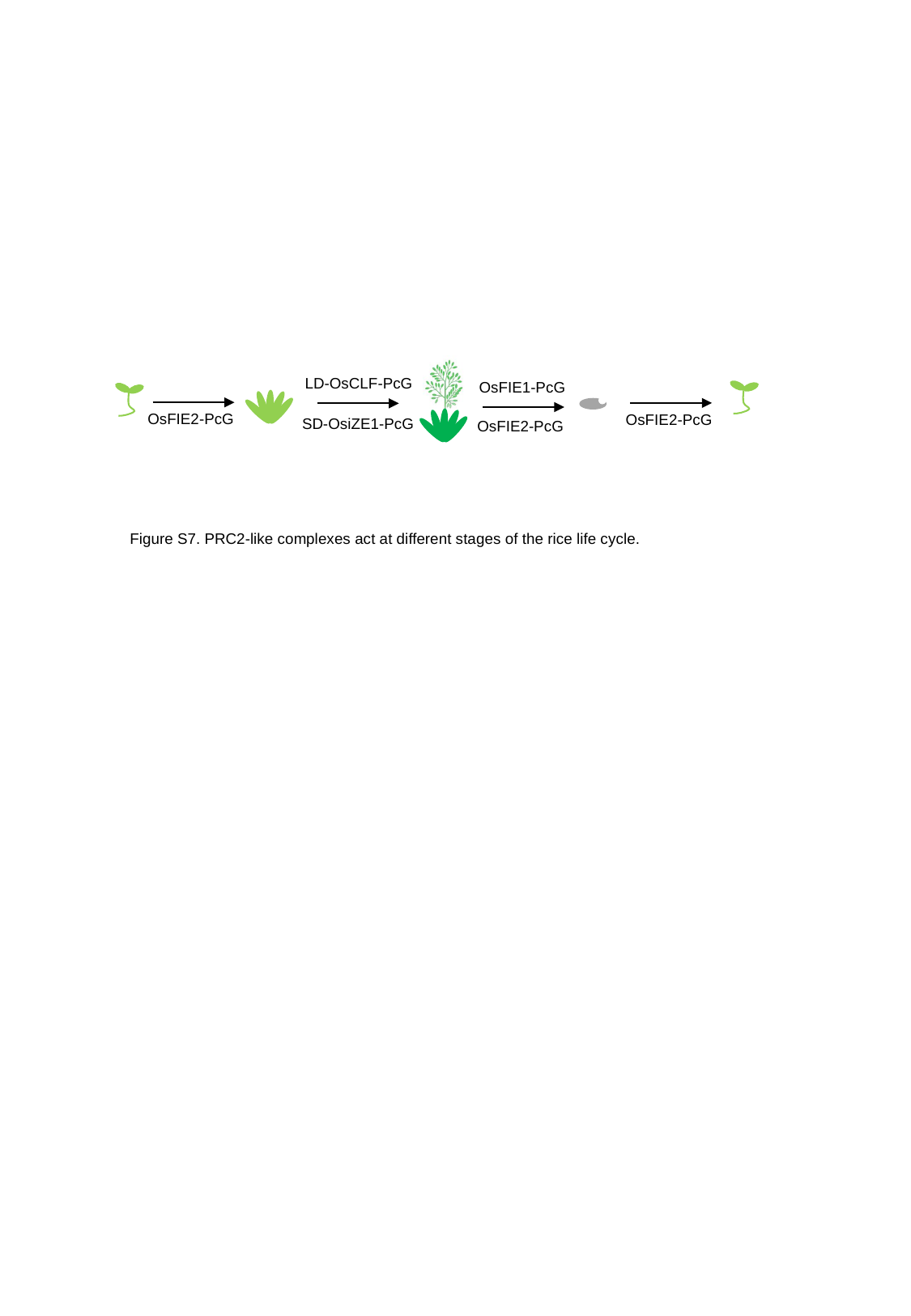

LD-OsCLF-PcG
OsFIE1-PcG
OsFIE2-PcG
OsFIE2-PcG
SD-OsiZE1-PcG
OsFIE2-PcG
Figure S7. PRC2-like complexes act at different stages of the rice life cycle.

## Slide 8
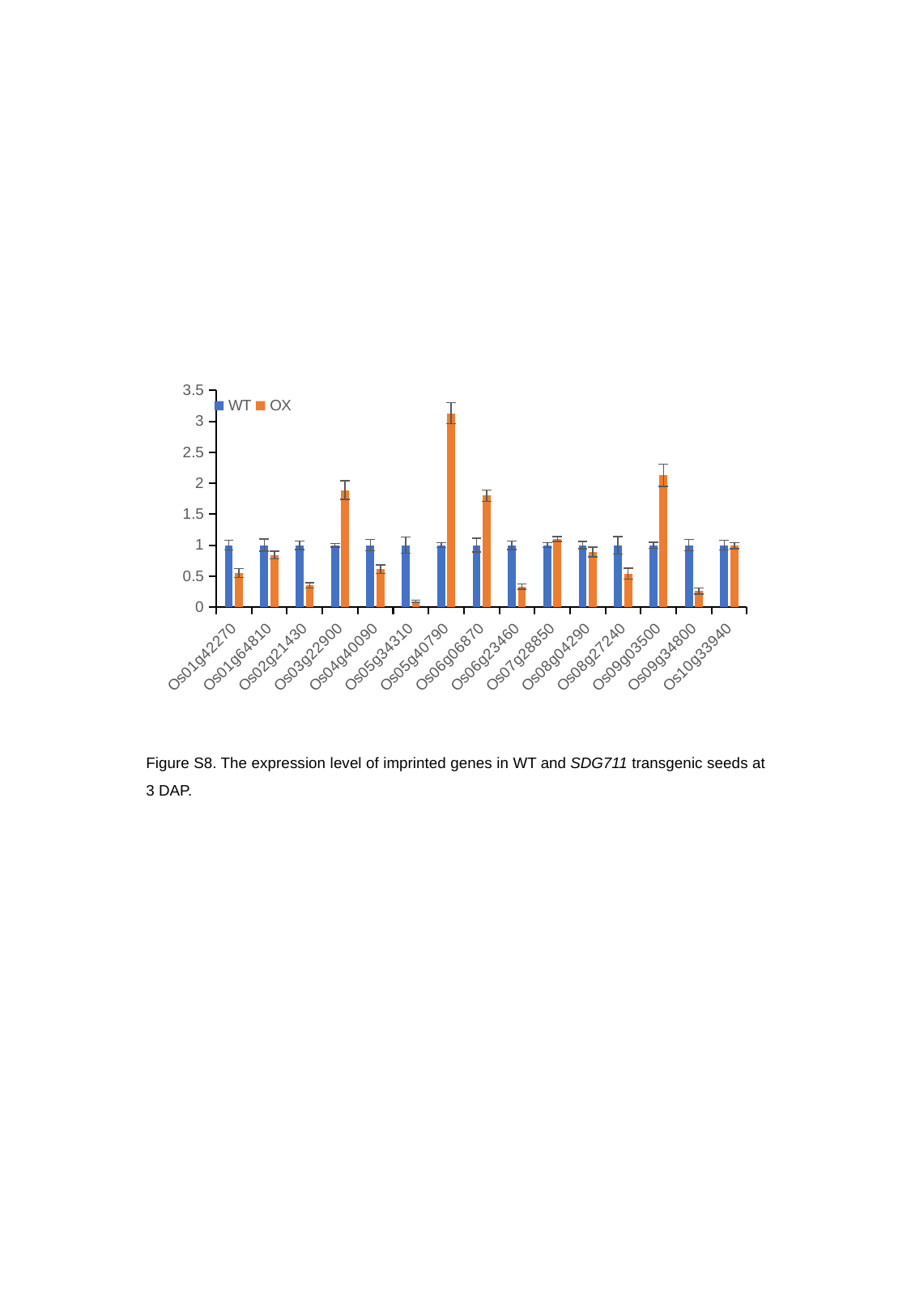

### Chart
| Category | WT | OX |
|---|---|---|
| Os01g42270 | 1.0 | 0.55 |
| Os01g64810 | 1.0 | 0.84 |
| Os02g21430 | 1.0 | 0.35 |
| Os03g22900 | 1.0 | 1.89 |
| Os04g40090 | 1.0 | 0.61 |
| Os05g34310 | 1.0 | 0.09 |
| Os05g40790 | 1.0 | 3.13 |
| Os06g06870 | 1.0 | 1.8 |
| Os06g23460 | 1.0 | 0.33 |
| Os07g28850 | 1.0 | 1.1 |
| Os08g04290 | 1.0 | 0.89 |
| Os08g27240 | 1.0 | 0.54 |
| Os09g03500 | 1.0 | 2.13 |
| Os09g34800 | 1.0 | 0.26 |
| Os10g33940 | 1.0 | 0.99 |Figure S8. The expression level of imprinted genes in WT and SDG711 transgenic seeds at 3 DAP.

## Slide 9
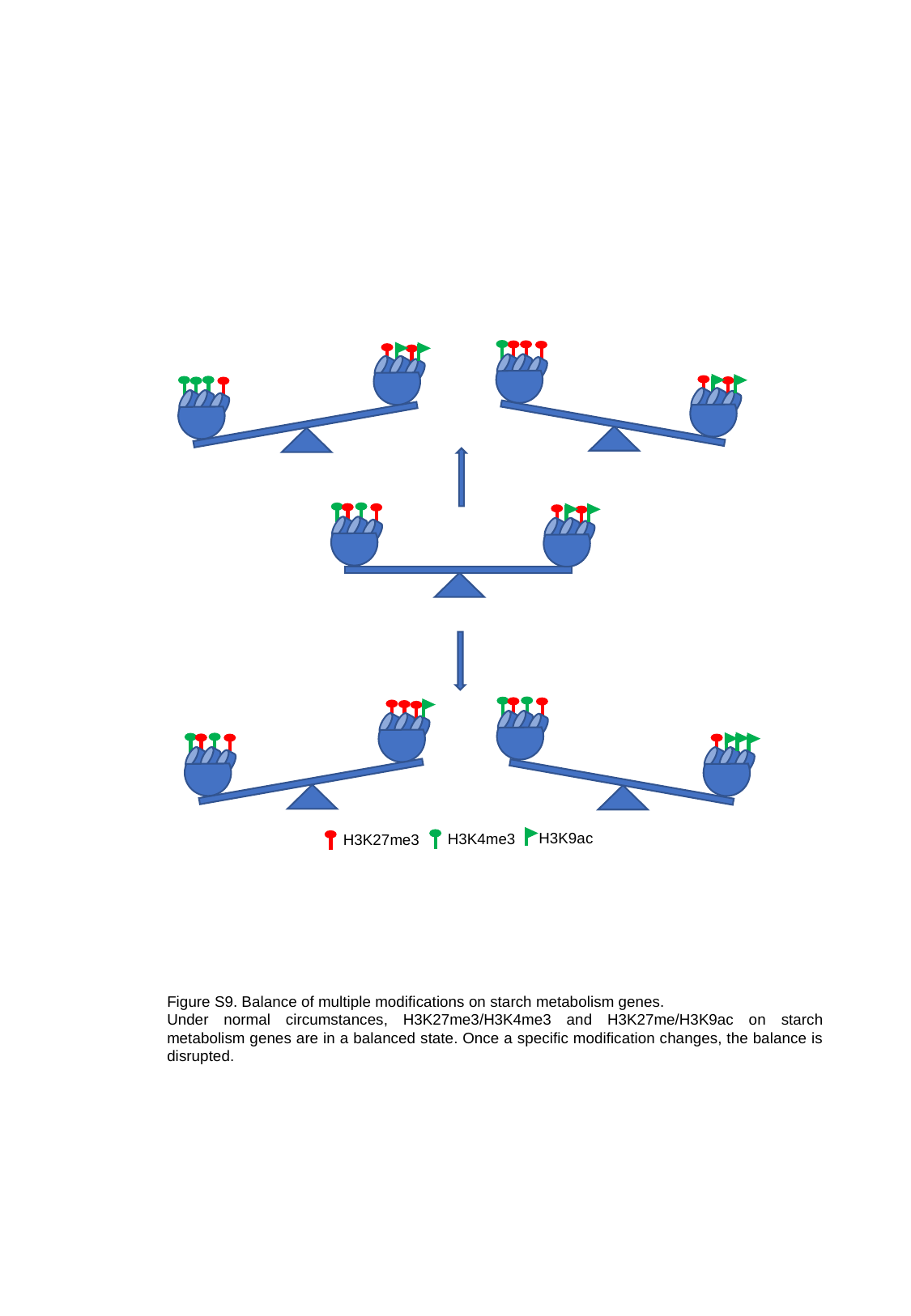

H3K9ac
H3K4me3
H3K27me3
Figure S9. Balance of multiple modifications on starch metabolism genes.
Under normal circumstances, H3K27me3/H3K4me3 and H3K27me/H3K9ac on starch metabolism genes are in a balanced state. Once a specific modification changes, the balance is disrupted.
