# Supplementary material for: SDG711 Is Involved in Rice Seed Development through Regulation of Starch Metabolism Gene Expression in Coordination with Other Histone Modifications
Source: Rice (N Y). 2021 Mar 5;14:25. doi: 10.1186/s12284-021-00467-y (PMC7936014; doi:10.1186/s12284-021-00467-y)
Supplement: Supplementary file 2 — Additional file 2: Table S1. Oligonucleotides primers used in the study. [file 12284_2021_467_MOESM2_ESM.docx]

**Supplemental Table 1. Oligonucleotides primers used in the study.**

| Primers for quantitative RT-PCR | Primer sequence | |
| --- | --- | --- |
| Actin-QF | TGAAGATCAAGGTGGTGGCAC | |
| Actin-QR | TGCTGGACCCGACTCATCATA | |
| RSR1-QF | GATAGCGGCTCCCTTGGC | |
| RSR1-QR | TGGCTTCTTTCCTTTTTATCAA | |
| OsAGPS1-QF | GTGCCACTTAAAGGCACCATT | |
| OsAGPS1-QR | CCCACATTTCAGACACGGTTT | |
| OsAGPS2a-QF | ACTCCAAGAGCTCGCAGACC | |
| OsAGPS2a-QR | GCCTGTAGTTGGCACCCAGA | |
| OsAGPS2b-QF | AACAATCGAAGCGCGAGAAA | |
| OsAGPS2b-QR | GCCTGTAGTTGGCACCCAGA | |
| OsAGPL1-QF | GGAAGACGGATGATCGAGAAAG | |
| OsAGPL1-QR | CACATGAGATGCACCAACGA | |
| OsAGPL2-QF | AGTTCGATTCAAGACGGATAGC | |
| OsAGPL2-QR | CGACTTCCACAGGCAGCTTATT | |
| OsAGPL3-QF | AAGCCAGCCATGACCATTTG | |
| OsAGPL3-QR | CACACGGTAGATTCACGAGACAA | |
| OsAGPL4-QF | TCAACGTCGATGCAGCAAAT | |
| OsAGPL4-QR | ATCCCTCAGTTCCTAGCCTCATT | |
| OsSSI-QF | GGGCCTTCATGGATCAACC | |
| OsSSI-QR | CCGCTTCAAGCATCCTCATC | |
| OsSSIIa-QF | GCTTCCGGTTTGTGTGTTCA | |
| OsSSIIa-QR | CTTAATACTCCCTCAACTCCACCAT | |
| OsSSIIb-QF | TAGGAGCAACGGTGGAAGTGA | |
| OsSSIIb-QR | GTGAACGTGAGTACGTGACCAAT | |
| OsSSIIc-QF | GACCGAAATGCCTTTTTCTCG | |
| OsSSIIc-QR | GGGCTTGGAGCCTCTCCTTA | |
| OsSSIIIa-QF | GCCTGCCCTGGACTACATTG | |
| OsSSIIIa-QR | GCAAACATATGTACACGGTTCTGG | |
| OsSSIIIb-QF | ATTCCGCTCGCAAGAACTGA | |
| OsSSIIIb-QR | CAACCGCAGGATAACGGAAA | |
| OsSSIVa-QF | GGGAGCGGCTCAAACATAAA | |
| OsSSIVa-QR | CCGTGCACTGACTGCAAAAT | |
| OsSSIVb-QF | ATGCAGGAAGCCGAGATGTT | |
| OsSSIVb-QR | ACGACAATGGGTGCCAAGAT | |
| OsGBSSI-QF | AACGTGGCTGCTCCTTGAA | |
| OsGBSSI-QR | TTGGCAATAAGCCACACACA | |
| OsGBSSII-QF | AGGCATCGAGGGTGAGGAG | |
| OsGBSSII-QR | CCATCTGGCCCACATCTCTA | |
| OsBEI-QF | TGGCCATGGAAGAGTTGGC | |
| OsBEI-QR | CAGAAGCAACTGCTCCACC | |
| OsBEIIa-QF | GCCAATGCCAGGAAGATGA | |
| OsBEIIa-QR | GCGCAACATAGGATGGGTTT | |
| OsBEIIb-QF | ATGCTAGAGTTTGACCGC | |
| OsBEIIb-QR | AGTGTGATGGATCCTGCC | |
| OsISA1-QF | TGCTCAGCTACTCCTCCATCATC | |
| OsISA1-QR | AGGACCGCACAACTTCAACATA | |
| OsISA2-QF | TAGAGGTCCTCTTGGAGG | |
| OsISA2-QR | AATCAGCTTCTGAGTCACCG | |
| OsISA3-QF | ACAGCTTGAGACACTGGGTTGAG | |
| OsISA3-QR | GCATCAAGAGGACAACCATCTG | |
| OsPUL-QF | ACCTTTCTTCCATGCTGG | |
| OsPUL-QR | CAAAGGTCTGAAAGATGGG | |
| OsAmy1A | GATACGACGTCGAACACCTC | |
| OsAmy1A | CGGATCGGATACAGCTCGTTG | |
| OsAmy1B | CCTTTCGGTCCTCATCGTCC | |
| OsAmy1B | TTGTACCACCCGCCATTCTC | |
| OsAmy1C | TATCATGGAGGCTGACAGCG | |
| OsAmy1C | GCTAATTGTGCCTCTCCACC | |
| OsAmy2A | GCCGATCATCGCACCTCTTC | |
| OsAmy2A | CGATCCCACATATCAGTGACG | |
| OsAmy3A | GAGGGTCATCACCAAGATCG | |
| OsAmy3A | TGTGTAGCTAGCTTGCGAGC | |
| OsAmy3B | GATTGGGACACGGTATGACG | |
| OsAmy3B | CTGCAGGAACTCTGAGACCG | |
| OsAmy3D | GTAGGCAGGCTCTCTAGCCT | |
| OsAmy3D | CCAACGGTTACAAACTGCGTGA | |
| OsAmy3E | GAAGGAAGGCCTCAGGGTTC | |
| OsAmy3E | GCTCGTACACATCTCGCAGCA | |
| OsAmy4A | TCGCCTGAAGGATACATGCC | |
| OsAmy4A | GATTCAGGACGGCATCACCA | |
| OsAmy5A | TCAGGGTCATTGGCCATTCC | |
| OsAmy5A | TGGGGATGCCAGGATGAGTA | |
| OsBmy1 | ACCTCTGAACGGCGAAAACT | |
| OsBmy1 | GCCACCTGAGTAGAGCCTTG | |
| OsBmy2 | AGAGGGCATACGTGCAGAAC | |
| OsBmy2 | TCCACTATCCCCCACCAACA | |
| OsBmy3 | AGAAGAACGGGCTCAAGGTG | |
| OsBmy3 | TCCTCCAACACCCATTTCGG | |
| OsBmy4 | GACATCCTCTTCGCCGACC | |
| OsBmy4 | GTGACAGTCGAGCCCAAGAA | |
| OsBmy5 | TGAGAGCGAACAGGCTTCTC | |
| OsBmy5 | ATGACGTAGACGGGGACGTA | |
| OsBmy6 | CGACATCGTCAACATCCCGA | |
| OsBmy6 | CCACCACGGTTGGTGTAGAA | |
| OsBmy7 | CAGGACCTCTTCGACTCCAC | |
| OsBmy7 | CTCCAGCATGTACCTGTCGT | |
| OsBmy8 | CCCCGTCTATGTCATGCTCC | |
| OsBmy8 | AATCGACCATGACCCCATCG | |
| OsBmy9 | CGGTACAACTTCGACGGCTA | |
| OsBmy9 | CGAGGTCGTTGTCCTTCTCC | |
| OsBmy10 | ACTGCGCCTACTCAAAGACC | |
| OsBmy10 | GATTCGGTTCGTGATTGCGG | |
| Os01g42270 | GACCAGGGAGTGGGGTAGAT | |
| Os01g42270 | GGAGCGAGTACACCCGTATG | |
| Os01g64810 | AAGCGTTCGTGAGGTTTTGC | |
| Os01g64810 | TCGCATCCGTTATCTCCACG | |
| Os02g21430 | CATATGGGCATGGGGAGGTC | |
| Os02g21430 | GAACAATGTCCTGGACGGGT | |
| Os03g22900 | TGCGATCGTCTGTAGAAGCC | |
| Os03g22900 | GCCATGCAGACCACAATCAC | |
| Os04g40090 | AATGCCGCGTTCTTGGTTTG | |
| Os04g40090 | TGCGGACCATTCACAAAAGC | |
| Os05g34310 | AATAGTGTGCGCCCTAGTCG | |
| Os05g34310 | GCACTGTAACCGTGAGACGA | |
| Os05g40790 | TATTGGCTACCCATGCCCAC | |
| Os05g40790 | GGCTGAGTTCTTCACCCCTC | |
| Os06g06870 | CCTACCGTGGCCAGTGAAAT | |
| Os06g06870 | TATCGCATCCATTGCTCCCC | |
| Os06g23460 | GATGAGGGCCGAGACCAAAA | |
| Os06g23460 | TCCGACAACGATACACACCG | |
| Os07g28850 | CCATTCCCATCTCCTCGTCG | |
| Os07g28850 | GATGATCGTGCGGAACAAGC | |
| Os08g04290 | CGAGTCGGGAGCGAGAATAC | |
| Os08g04290 | GGTGGCGAAGATGTCGTAGT | |
| Os08g27240 | CGTCCGCCTTTTCCTACCAT | |
| Os08g27240 | CCTGCAGACACTCTAGGGCT | |
| Os09g03500 | TTCACTTCCTCGTTGGGGTG | |
| Os09g03500 | CTATCGGCACTGAAAGGGCA | |
| Os09g34800 | GGTGACAAGAGGGTTCCCAG | |
| Os09g34800 | ACCCTCCCCTCCTCTTTGTT | |
| Os10g33940 | CTGTTCGGTTTTTGCGGTGT | |
| Os10g33940 | CTGCAACAGAAACGGTGGTG | |
| Primers for ChIP analysis | | Primer sequence |
| ChIP-OsSSI-F1 | CGCCTCTCCCTCGCTTAAAA | |
| ChIP-OsSSI-R1 | CACGAGGAAGGTCGGCAG | |
| ChIP-OsSSI-F2 | TGCCGAGATACATGAACGGG | |
| ChIP-OsSSI-R2 | TCTCCGCCAAAGCATGGAAT | |
| ChIP-OsSSIIa-F1 | CACGCTTTACAGGTGGCTCC | |
| ChIP-OsSSIIa-R1 | GTGCTTTGCTTTCGTCCCG | |
| ChIP-OsSSIIa-F2 | AAGAACGGAGCGCTTCTCAG | |
| ChIP-OsSSIIa-R2 | GCTGGGAGCTTCGTTATGGT | |
| ChIP-OsAGPL3-F1 | CCGTTTCTCTCTCCGGCATT | |
| ChIP-OsAGPL3-R1 | CGCCATCACTACGCGTCTAC | |
| ChIP-OsAGPL3-F2 | TGCCGATGAGCAACTGCATA | |
| ChIP-OsAGPL3-R2 | GCACCTCGACGAATCCATCT | |
| ChIP-OsGBSSI-F1 | CGCCACTGGAGAAACAGATCA | |
| ChIP-OsGBSSI-R1 | TCCTTTGACCAACTCGGCTAC | |
| ChIP-OsGBSSI-F2 | GACCGTCGTCGTCTTCAACT | |
| ChIP-OsGBSSI-R2 | CCAAGCGTCCTTGTACTGGT | |
| ChIP-OsBE1-F1 | GCTGCACAAGCTTTTCCGTG | |
| ChIP-OsBE1-R1 | GTGGAGGCGACTCCCATTTC | |
| ChIP-OsBE1-F2 | CTGACGTGTGGTGTCTGTCT | |
| ChIP-OsBE1-R2 | GGTGGAGGTACTGGATGGTC | |
| ChIP-OsISA2-F1 | CCGCTCGGCATGTCTATCTC | |
| ChIP-OsISA2-R1 | AACGGCATTGGCATTCTTGC | |
| ChIP-OsISA2-F2 | TGGGCTGAGGTGAACACAAG | |
| ChIP-OsISA2-R2 | GCCCTCTGGTTGAGAACAGG | |
| ChIP-OsAmy1C-F1 | GCAAACGCTTCTTGTCCCTG | |
| ChIP-OsAmy1C-R1 | TTGTACCACCCGCCATTCTC | |
| ChIP-OsAmy1C-F2 | CAGGGCTTCAACTGGGAGTC | |
| ChIP-OsAmy1C-R2 | GGAAGCGTCCAGATCGTACA | |
| ChIP-OsAmy3B-F1 | ACATCGACCACCTCAACACG | |
| ChIP-OsAmy3B-R1 | TATCCCTTGGCGAAGTCGAG | |
| ChIP-OsAmy3B-F2 | TCGCCGAGATATGGAGCAAC | |
| ChIP-OsAmy3B-R2 | CCCTTGGTCGTGAAGTCGAA | |
| ChIP-OsBmy4-F1 | CCAAACCAAACCCAAACGAGA | |
| ChIP-OsBmy4-R1 | CACTCGATCGCGACTTTGCT | |
| ChIP-OsBmy4-F2 | TCCATGACTTCTTGGGCTCG | |
| ChIP-OsBmy4-R2 | AGGCGGATACGAAGGGTACT | |
| ChIP-OsBmy9-F1 | CGGATCAAGGCTGTGTCGAT | |
| ChIP-OsBmy9-R1 | CCAACAAAACGCGGGAAACA | |
| ChIP-OsBmy9-F2 | GCCTTCCAATGCAACGACAG | |
| ChIP-OsBmy9-R2 | CAGGCTGCTACGCATGTACT | |
